# A Hematogenously Disseminated *Orientia tsutsugamsushi*-Infected Murine Model of Scrub Typhus

**DOI:** 10.1371/journal.pntd.0002966

**Published:** 2014-07-10

**Authors:** Thomas R. Shelite, Tais B. Saito, Nicole L. Mendell, Bin Gong, Guang Xu, Lynn Soong, Gustavo Valbuena, Donald H. Bouyer, David H. Walker

**Affiliations:** 1 Department of Pathology, Center for Biodefense and Emerging Infectious Diseases, Center for Tropical Diseases, Sealy Center for Vaccine Development, Institute of Human Infections and Immunity, The University of Texas Medical Branch, Galveston, Texas, United States of America; 2 Department of Microbiology and Immunology, The University of Texas Medical Branch, Galveston, Texas, United States of America; University of California San Diego School of Medicine, United States of America

## Abstract

*Orientia tsutsugamushi*, the etiologic agent of scrub typhus, is a mite-borne rickettsia transmitted by the parasitic larval stage of trombiculid mites. Approximately one-third of the world's population is at risk of infection with *Orientia tsutsugamushi*, emphasizing its importance in global health. In order to study scrub typhus, *Orientia tsutsugamushi* Karp strain has been used extensively in mouse studies with various inoculation strategies and little success in inducing disease progression similar to that of human scrub typhus. The objective of this project was to develop a disease model with pathology and target cells similar to those of severe human scrub typhus. This study reports an intravenous infection model of scrub typhus in C57BL/6 mice. This mouse strain was susceptible to intravenous challenge, and lethal infection occurred after intravenous inoculation of 1.25×10^6^ focus (FFU) forming units. Signs of illness in lethally infected mice appeared on day 6 with death occurring ∼6 days later. Immunohistochemical staining for *Orientia* antigens demonstrated extensive endothelial infection, most notably in the lungs and brain. Histopathological analysis revealed cerebral perivascular, lymphohistiocytic infiltrates, focal hemorrhages, meningoencephalitis, and interstitial pneumonia. Disseminated infection of endothelial cells with *Orientia* in C57BL/6 mice resulted in pathology resembling that of human scrub typhus. The use of this model will allow detailed characterization of the mechanisms of immunity to and pathogenesis of *O. tsutsugamushi* infection.

## Author Summary

Scrub typhus is a disease found in Southeast Asia that infects over 1 million people each year. This disease is caused by the intracellular pathogen *Orientia tsutsugamushi* transmitted by the bite of chigger mites. Scrub typhus is characterized by pulmonary disease and in severe cases, multiorgan system failure. The current research model utilizes an intraperitoneal route of inoculation of mice to study the host response to *Orientia* infection. Infection via this route results in severe peritonitis that does not occur in human scrub typhus. The development of animal models that accurately portray human disease is an important step toward understanding and managing disease. In this manuscript we describe a new mouse model that results in scrub typhus-like pathology following intravenous inoculation of mice. This model presents dose-dependent mortality with scrub typhus-like pathology that parallels human disease. Utilization of this model will provide a valuable research tool for characterizing the immune response and pathogenesis induced by *O. tsutsugamushi* allowing development of better treatment and an effective vaccine.

## Introduction

*Orientia tsutsugamushi*, a gram-negative obligately intracellular coccobacillus, is the etiologic agent of scrub typhus [1]. Scrub typhus is a serious public health problem in Asia, northern Australia, and islands of the western Pacific and Indian Oceans including Korea, Japan, China, Taiwan, Indonesia, India and Thailand, and threatens one billion persons globally and causes illness in one million people each year [2]. One to two weeks after being fed upon by an infected larval *Leptotrombidium* mite, patients exhibit signs of infection such as an inoculation site eschar followed by lymphadenopathy, fever and rash accompanied by non-specific flu-like symptoms. Without appropriate treatment, scrub typhus can cause severe multiorgan failure with a case fatality rate of 7–15%. Doxycycline, azithromycin, rifampicin, and chloramphenicol are the antibiotics used to treat *Orientia* infection and are effective if begun early in the disease course [3]. However, misdiagnosis, inappropriate antibiotic treatment, and antibiotic failures have occurred, supporting the need for a vaccine [4]. Antigenic heterogeneity and immunity that wanes after infection leading to reinfections are major obstacles to vaccine development [5], [6]. The current reemergence of scrub typhus further emphasizes the need for the development of a vaccine, which requires appropriate animal models for determining mechanisms of immunity, candidate vaccine efficacy, and correlates of immune protection.

During *Orientia* infection, the bacteria infect endothelial cells, macrophages, cardiac myocytes, and dendritic cells [7], [8]. Similar to most of the bacteria belonging to the family Rickettsiaceae, *Orientia* exhibits endothelial tropism. As such, scrub typhus is a disseminated endothelial infection that affects all organs. Primary characteristics of fatal scrub typhus pathology include diffuse interstitial pneumonia, hepatic lesions, meningoencephalitis, and coagulation disorders [7], [9]–[11]. However, current murine models fail to reproduce the pathology of human scrub typhus.

The lack of an appropriate animal model of scrub typhus has fundamentally impeded progress in this field. The currently available model, first employed more than 60 years ago, uses intraperitoneal inoculation of *O. tsutsugamushi* into mice, which establishes infection in the peritoneal cavity [12], [13]. Continuous proliferation of *O. tsutsugamushi* in peritoneal macrophages and mesothelial cells, enlargement of spleen and liver, and severe peritonitis occur in intraperitoneally inoculated mice [14]–[16]. However, the pathology of *O. tsutsugamushi* in human scrub typhus consists of disseminated endothelial injury and lymphohistiocytic vasculitis as the basis for interstitial pneumonitis, hepatic damage, and encephalitis [7], [11], [17], which differ considerably from the histopathologic lesions of intraperitoneally inoculated mice. Based on previous histopathological and immunohistochemical observations of scrub typhus in humans and our experience with models of rickettsioses, we hypothesized that inoculation of mice via the intravenous route would result in scrub typhus-like pathology. Our studies have determined reproducibly lethal and sublethal doses as well as the LD_50_ of intravenously inoculated *O. tsutsugamushi* in mice. We employed these doses to compare the histopathology with those of mice challenged using the classic intraperitoneal model. All parameters, including bacterial loads and histopathology of lungs, spleen, liver and brain confirmed that the intravenously inoculated mice provide a model of scrub typhus that closely resembles the human disease.

## Materials and Methods

### Ethics statement

C57BL/6 (B6) and C3H/HeN (C3H) mice were purchased from Harlan Laboratories, Houston, TX. Age- and gender-matched, 8–12 week old mice were used in all studies. Experimentally infected mice were housed in an animal biosafety level 3 facility, and all experiments and procedures were approved by the Institutional Animal Care and Use Committee (IACUC) of the University of Texas Medical Branch (Protocol: 9008082), Galveston in accordance with Guidelines for Biosafety in Microbiological and Biomedical Laboratories. UTMB operates to comply with the USDA Animal Welfare Act (Public Law 89-544), the Health Research Extension Act of 1985 (Public Law 99-158), the Public Health Service Policy on Humane Care and Use of Laboratory Animals, and the NAS Guide for the Care and Use of Laboratory Animals (ISBN-13). UTMB is a registered Research Facility under the Animal Welfare Act, and has a current assurance on file with the Office of Laboratory Animal Welfare, in compliance with NIH Policy.

### Bacterial culture

*Orientia tsutsugamushi* Karp strain was cultivated in Vero E6 cells or serially passaged in 8–12 week old female C57BL/6 mice (Harlan Laboratories, Houston, TX). For cultivation in Vero cells, bacteria were inoculated onto confluent monolayers in T150 cell culture flasks and gently rocked for two hours at 34°C, at the end of which Dulbecco's Modified Eagles Medium (DMEM, Gibco) with 1% fetal bovine serum (FBS) and 1% HEPES buffer were added. Cells were observed for cytopathic effect, which usually occurred at 14–21 days. When areas of rounded or floating cells were observed throughout the flask, a smear was prepared, and the level of infection was assessed either by Dif-Quik (Fisher Scientific, Kalamazoo, MI) or immunofluorescence staining. When the flask reached 80–90% of cells infected, the cells were removed and seeded onto fresh Vero cell monolayers. This process was repeated for a total of six passages. Infected flasks were harvested by scraping, and cell suspensions were collected in Oakridge high speed centrifugation bottles and centrifuged at 22,000× g for 45 minutes at 4°C. The pellet was resuspended in sucrose-phosphate-glutamate (SPG) buffer (0.218 M sucrose, 3.8 mM KH_2_PO4, 7.2 mM KH_2_PO4, 4.9 mM monosodium L-glutamic acid, pH 7.0) and transferred to a 50 mL conical tube containing 5 mL of sterile glass beads. The conical tubes were gently vortexed at 10 sec intervals to release the intracellular bacteria and placed on ice. The tubes were then centrifuged at 700× g to pellet cell debris, and the supernatant was collected. The tubes were then centrifuged at 22,000× g for 45 minutes to pellet cell-free bacteria. Pellets were resuspended in SPG buffer and stored at −80°C until used. Preliminary studies were conducted using cell cultured orientiae.

Animal passages were performed to rapidly produce high titered oriential stocks from infected tissues. Two groups of four 8–12 week old female C57BL/6 mice were inoculated intravenously with 1.25×10^6^ focus forming units (FFU) of *Orientia* cultivated in Vero E6 cells. When the animals exhibited signs of illness, i.e., hunched posture, lethargy, and ruffled fur, usually at six days post infection, they were euthanized, and the liver and lungs aseptically collected and placed in DMEM. Organ-specific pools were homogenized using a 7 mL glass Dounce apparatus. Homogenized samples were then rinsed with cold SPG buffer and placed in a 50 mL conical tube and centrifuged at 700× g for 10 minutes at 4°C to pellet the tissue debris. Supernatant fluid was collected and placed on ice. The tissue pellets were resuspended in 5 mL of SPG buffer, homogenized and centrifuged as above. Organ-specific supernatants were pooled on ice and then centrifuged at 22,000× g for 45 minutes at 4°C in a Beckman high speed centrifuge. The pellets were resuspended in 10 mL of SPG buffer, aliquoted, and stored at −80°C.

### Focus forming assays (FFA)

It takes greater than 15 days for plaques to form in *O. tsutsugamushi*-infected monolayers [18, personal observation]. Thus, in order to quantitate the number of viable bacteria in a timely manner, a focus forming assay was used [19]. Vero E6 cells in DMEM with 1% FBS and 1% HEPES were seeded onto 12-well plates and allowed to attach overnight at 37°C in a 5% CO_2_ atmosphere. Once the cells were confluent, serial 10-fold dilutions of oriential stocks were prepared, and 200 µL aliquots were seeded onto the confluent monolayers in triplicate. The inoculated plates were centrifuged for 5 minutes at 700× g to facilitate bacterial attachment and then incubated for two hours at 37°C in 5% CO_2_. After two hours, the wells were rinsed three times with warm Dulbecco's PBS (Cellgro, Manassas, VA) with calcium and magnesium to remove extracellular non-viable bacteria. The wells were then overlaid with DMEM containing 1% FBS, 0.5% sterile methylcellulose, and 2 µg cyclohexamide and incubated at 34°C for 5 days. After 5 days, the overlay was aspirated and the monolayers gently rinsed as above. The monolayers were fixed in methanol for 30 minutes at 4°C, after which the methanol was removed and the wells rinsed as above. Wells were blocked using PBS with 1% BSA for 30 minutes at room temperature. Blocking buffer was then removed and the wells washed three times with 0.5% Tween-20 in PBS. An aliquot of primary polyclonal rabbit anti-*O. tsutsugamushi* Karp strain antibody (1∶500 dilution) was added to each well and incubated at room temperature for 30 minutes. The primary antibody was removed and wells washed as above. Alexa-594 goat anti-rabbit IgG (Invitrogen, Carlsbad CA) diluted 1∶1,000 was added to each well, incubated for 30 minutes and then washed as above. Wells were examined using an inverted fluorescent microscope. Wells containing 10–100 foci of cells infected with *Orientia* were counted, and the concentration of focus-forming units was calculated.

### Mouse strain comparison

A large number of mouse strains has been shown to be susceptible to intraperitoneal inoculation of *O. tsutsugamushi* Karp strain including C57BL/6 and C3H/HeN mice [20], but very few data exist describing susceptibility via intravenous challenge. Our laboratory has extensive experience with mouse model development for rickettsioses with these mouse strains [21]–[24]. In this study, both C57BL/6 and C3H/HeN mice strains were compared for susceptibility to *Orientia* infection via intravenous challenge. To determine the infectivity of the cell culture stock, intraperitoneally (i.p.) inoculated animals were studied in parallel with intravenously (i.v.) inoculated animals to ensure infectivity of the *Orientia*. Animals were challenged with 2.5×10^6^ or 1.25×10^6^ organisms and were observed daily for signs of illness for 28 days post infection (dpi) or until animals became moribund.

### Characterization of lethal and sublethal infections of mice with *Orientia tsutsugamushi*

*Orientia tsutsugamushi* Karp strain, passaged and maintained as described above, was diluted in PBS, and the bacteria were injected i.v. through the tail vein or i.p. in a volume of 200 µL. Control mice were inoculated with 200 µL of similarly prepared material from uninfected cells or tissue diluted in PBS. Animals were challenged with 1.25×10^6^, 1.25×10^5^, and 1.25×10^4^ organisms either i.p. or i.v. All infected and non-infected animals were monitored for signs of illness and body weight measured daily until day 21. Mice were necropsied at 3, 6, 9, and 12 dpi or when moribund for lethally challenged animals and also at 15 dpi for sublethal i.v. challenge. Four randomly selected i.p. and i.v. inoculated animals were euthanized, and blood, brain, heart, kidney, liver, lung, lymph nodes, and spleen were collected for histopathology and blood, liver, lung, and spleen for bacterial load determination.

### Histopathology and immunohistochemistry

All tissues were fixed in 10% neutral buffered formalin and embedded in paraffin. Tissue sections (5 µm thickness) were stained with hematoxylin and eosin or processed for immunohistochemistry. Immunohistochemical staining was used to assess cellular distribution and intensity of *Orientia* infection in the organs of experimental animals. Sections were deparaffinized and rehydrated. The sections were placed on poly-L-lysine -coated slides and incubated at 70°C for 20 minutes, then rehydrated in water and treated with antigen retrieval solution. Antigen retrieval was accomplished by incubation in citrate buffer (pH = 6) at 98°C for 20 minutes followed by casein endogenous IgG blocking for 15 minutes to reduce possible species cross reactivity. Endogenous alkaline phosphatase activity was quenched by incubation with Levamisole (Sigma Aldrich, St. Louis, MO) for 15 minutes and slides rinsed in deionized water. Nonspecific binding of antibody was blocked by incubating sections with normal goat serum and avidin blocking reagent (Vector Laboratories, Burlingame, CA) mixture (1∶10) for 30 minutes. Sections then were incubated for 2 hours with polyclonal rabbit anti-*O. tsutsugamushi* Karp strain antibody (dilution: 1∶500), followed by incubation for 30 minutes with biotinylated anti-rabbit IgG (1∶2000, Vector Laboratories, Burlingame, CA). Signals were detected by the labeled streptavidin-biotin method with an UltraVision Alk-Phos kit (Thermo Scientific, Waltham, MA). Vector Red Alkaline Phosphatase substrate (Vector Laboratories, Burlingame, CA) was used as chromogen, and counterstaining was performed with hematoxylin. Reagent negative controls consisted of samples in which primary antibody was replaced with normal rabbit IgG. Sections were mounted in Permount.

### Bacterial load determination

Bacterial loads were assessed by quantitative real-time PCR [25]. DNA was extracted using a DNeasy Kit (Qiagen, Gaithersburg, MD) from the tissue samples, and the bacterial load at each time point and for each organ sampled was determined by quantitative real-time PCR [25]. The 47 kDa gene was amplified using the primer pair OtsuF630 (5′-AACTGATTTTATTCAAACTAATGCTGCT-3′) and OtsuR747 (5′-TATGCCTGAGTAAGATACGTGAATGGAATT-3′) primers (IDT, Coralville, IA) and detected with the probe OtsuPr665 (5′-6FAM-TGGGTAGCTTTGGTGGACCGATGTTTAATCT-TAMRA) (Applied Biosystems, Foster City, CA). Bacterial loads were normalized to total nanogram (ng) of DNA per µL for the same sample and expressed as the number of 47 kDa gene copies per picogram (pg) of DNA.

### Blood cell counts

At the time points that animals were euthanized, blood samples were collected in K_2_EDTA-coated BD microtainer tubes (Becton Dickinson, Franklin Lakes, NJ) and blood cell counts performed using a 950FS HemaVet apparatus (Drew Scientific Inc., Waterbury, CT) that differentiates cell types by size and granularity in a 20 µL sample of whole blood.

### Transmission electron microscopy

Lung tissue from lethally infected animals was collected at 6 dpi and prepared for transmission electron microscopy. For ultrastructural analysis in ultrathin sections small pieces (∼1 mm^3^) of tissues were fixed for at least 1 hour in a mixture of 2.5% formaldehyde prepared from paraformaldehyde powder, and 0.1% glutaraldehyde in 0.05 M cacodylate buffer, pH 7.3, to which 0.03% picric acid and 0.03% CaCl_2_ were added. Then they were washed in 0.1 M cacodylate buffer and post-fixed in 1% OsO_4_ in 0.1 M cacodylate buffer, pH 7.3, for 1 hour, washed with distilled water and stained *en bloc* with 2% aqueous uranyl acetate for 20 min at 60°C. The samples were dehydrated in ethanol, processed through propylene oxide and embedded in Poly/Bed 812 (Polysciences, Warrington, PA). Semi-thin sections 1 µm thick were cut and stained with toluidine blue. Ultrathin sections were cut on Leica EM UC7 ultramicrotome (Leica Microsystems, Buffalo Grove, IL), stained with lead citrate and examined in a Philips 201 transmission electron microscope at 60 kV.

## Results

### Mouse strain comparison

B6 and C3H mice were compared for susceptibility, bacterial loads, and histopathology. To compare mouse strain susceptibility, i.p. inoculated animals were studied in parallel with i.v. inoculated animals. Inoculation of 10^3^ *Orientia* resulted in clinical illness (ruffled fur, hunched posture, and lethargy) in i.p. inoculated animals at 12–15 days post infection; in contrast, signs of illness at this dose in i.v. inoculated animals were mild, i.e., slightly ruffled fur with hunched posture but normal activity. Histopathologic examination demonstrated systemic lesions most prominently in the lungs and liver of animals inoculated i.v. with this dose of *O. tsutsugamushi*. Lethality was observed in the intravenous model using doses of both 2.5×10^6^ and 1.25×10^6^ organisms. C3H mice became moribund at 7–8 days post-inoculation (dpi) with either dose. The B6 mice that received 2.5×10^6^ organisms became moribund at 9–11 dpi; whereas those animals receiving 1.25×10^6^ organisms became moribund 10–13 dpi. Mice of both strains inoculated i.p. with either dose expired at 7–8 dpi. As both strains were susceptible all further studies were conducted with B6 mice only due to the availability of a variety of genetically modified strains on this background and the longer course similar to human scrub typhus observed in B6 mice.

### Characterization of high dose *Orientia* infection in C57BL/6 mice

**Figure 1:**
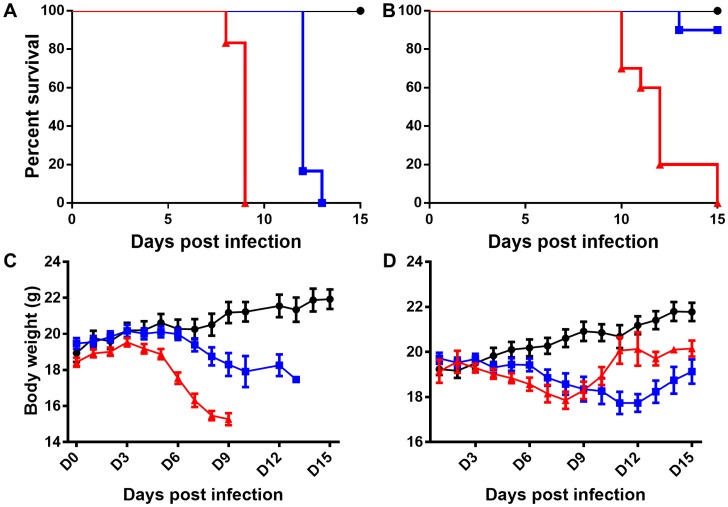
Survival and body weight change of mice inoculated with *O. tsutsugamushi* intraperitoneally or intravenously. Animals inoculated intraperitoneally with 1.25×10^6^ organisms (**A** and **C- red triangles**) began losing weight at 4 dpi that continued until death and expired by 9 dpi. Intravenously inoculated animals expired by 13 dpi with weight loss starting at 7 dpi and continuing until death (**A** and **C- blue boxes**). Animals inoculated i.p with 1.25×10^4^ organisms expired by 15 dpi losing weight until day 7 when most animals began to increase in body weight (**B** and **D- red triangles**). This late increase in body weight corresponded to an increase in accumulation of peritoneal fluid. Animals inoculated i.v. with the same dose became ill at 6–7 dpi, but only 1 of 10 animals expired with all animals losing weight until 12 dpi when signs of illness began to abate and animals appeared to be recovering (**B** and **D- blue boxes**). Uninfected controls are represented as solid black circles in all panels.

All mice challenged i.v. with 1.25×10^6^ bacteria expired by 13 dpi (**Figure 1A**), approximately half of the animals inoculated i.v. with 10^5^ *Orientia* expired between 13 and 15 dpi (data not shown), and 10% of mice challenged with 10^4^ *Orientia* expired by 15 dpi (**Figure 1B**). Intraperitoneal inoculations of all doses were uniformly lethal (**Figure 1A and B**). Controls were monitored until 21 dpi without morbidity.

**Disease progression of intraperitoneally inoculated mice:**

**Figure 2:**
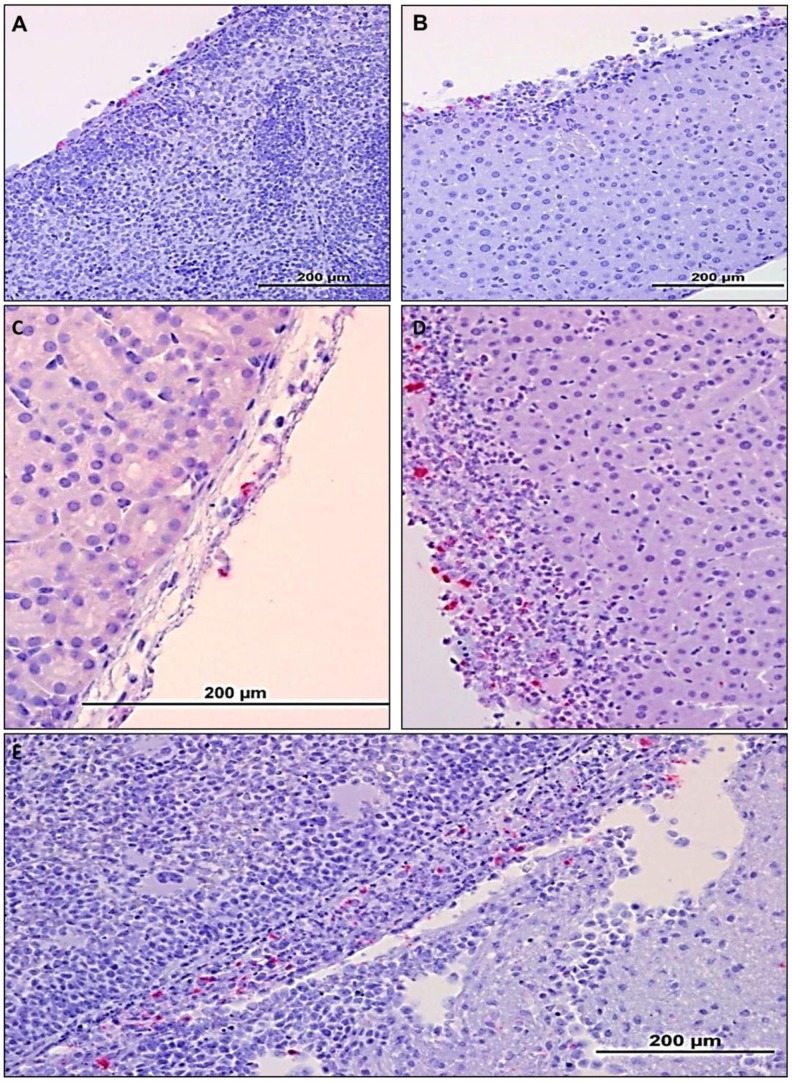
Histopathology in mice following 1.25×10^6^ *Orientia* intraperitoneal challenge at days 6 and 9 pi. On 6 dpi *Orientia* antigen was detected on the surface of the peritoneal organs (**A**, **B; 20×**). The spleen at this time was slightly enlarged with no other gross pathology (**A**). The surface of the liver at this time point revealed multifocal *Orientia* antigen and mesothelial hyperplasia. By 9 dpi bacterial antigen was detected on the surface of the renal capsule (**C-40×**) and in the hyperplastic inflamed surfaces of the liver (**D-40×**) and the spleen (**E-40×**).

At 3 dpi, there were no signs of illness except mild abdominal swelling. Overall activity was unchanged, and there was no weight change (**Figure 1C**). Mice manifested mesenteric lymphadenopathy and mild accumulation of fibrin-containing proteinaceous fluid in the peritoneal cavity causing the lobes of the liver to adhere to one another. Portions of the gastrointestinal tract were edematous and discolored. Neither histopathologic lesions nor *Orientia* antigen was detected on day 3 in mice inoculated i.p. At 6 dpi, mice inoculated i.p. had begun to lose weight (**Figure 1C** **-red triangles**) with narrowed eyes, severely hunched posture, and swollen abdomen. The animals' activity was diminished compared to uninfected controls. *Orientia* antigen was detected in endothelial cells in the lungs of these animals, but with minimal cellular response. Of particular interest were the moderate accumulation of peritoneal exudate and the extensive distribution of oriential antigen in cells on the peritoneal surfaces of all abdominal organs and mild-to-severe mesothelial hyperplasia (**Figure 2 A** and **B**). All animals were moribund or had expired by day 9; severe peritonitis was observed with accumulation of 2–4 mL of peritoneal exudate. *Orientia* antigen was detected in the lungs in association with vasculitis and interstitial pneumonia. These findings indicate that *Orientia* had eventually disseminated from the peritoneal cavity, but the most striking observation at this time was mesothelial hyperplasia and inflammation on the peritoneal surface of liver (**Figure 2D**) and spleen (**Figure 2E**) and the extensive *Orientia* infection of these cells. Proteinaceous material and infiltrating cells were also observed on the peritoneal surface of the spleen (**Figure 2E**). The cells overlying the kidney capsule (**Figure 2C**) were also infected.

**Disease progression of intravenously inoculated mice:**

**Figure 3:**
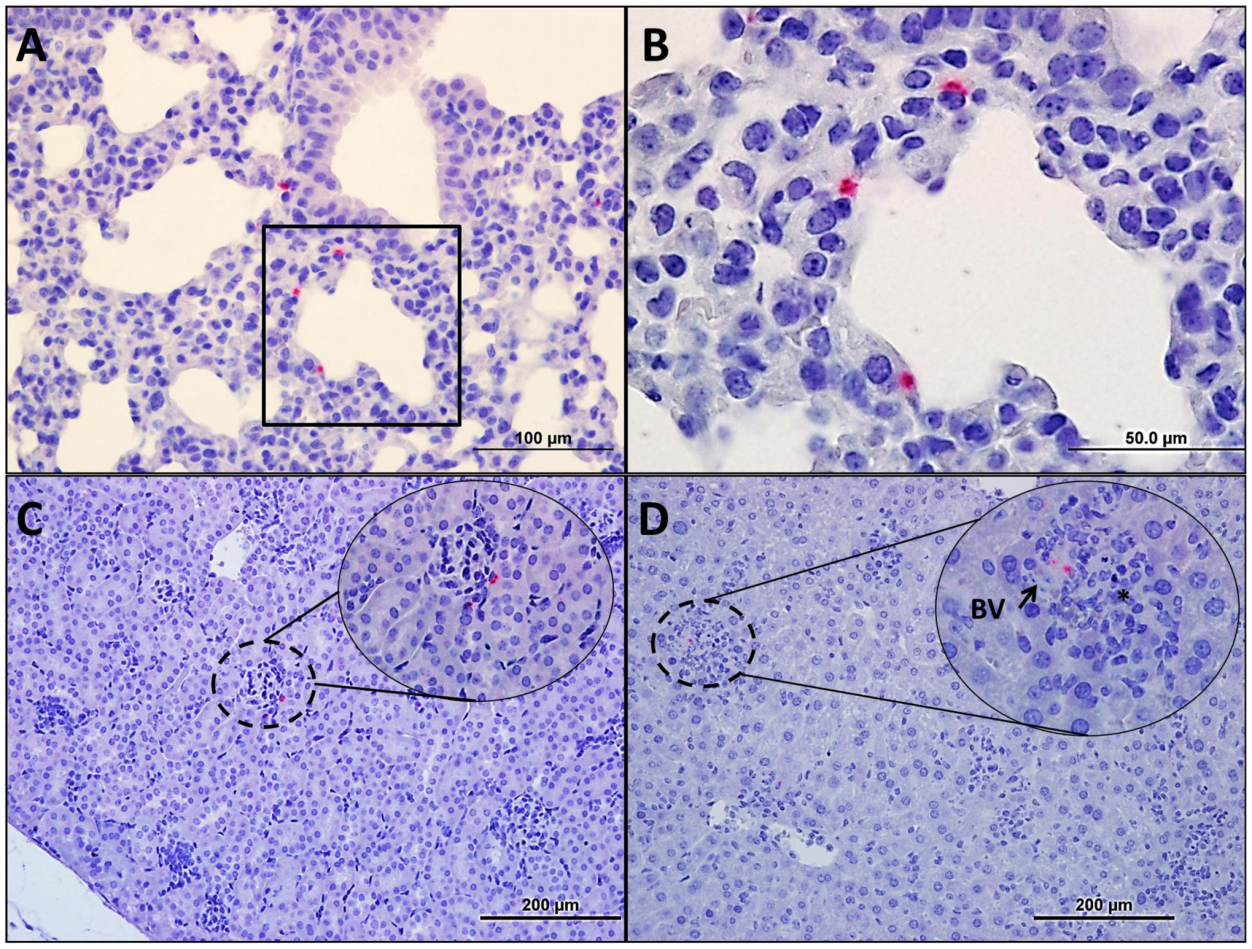
Histopathology in mice following 1.25×10^6^ *Orientia* intravenous challenge at 6 dpi. All organs, except brain, had detectable *Orientia* antigen (**hashed circles and insets B–E**). *Orientia* antigen in the lung (**A- 40×**, **B-100×**) was associated with vasculitis and interstitial pneumonia. Although antigen was detected in the kidney (**C-20×**; **inset-40×**) and spleen, no lesions were observed. Hepatic (**D-20×**; **inset-40×**) lesions increased in number and relative size and were often associated with blood vessels (**BV**). At this time, it was evident that systemic infection had been established with the majority of *Orientia* antigen present in endothelial locations.

**Figure 4:**
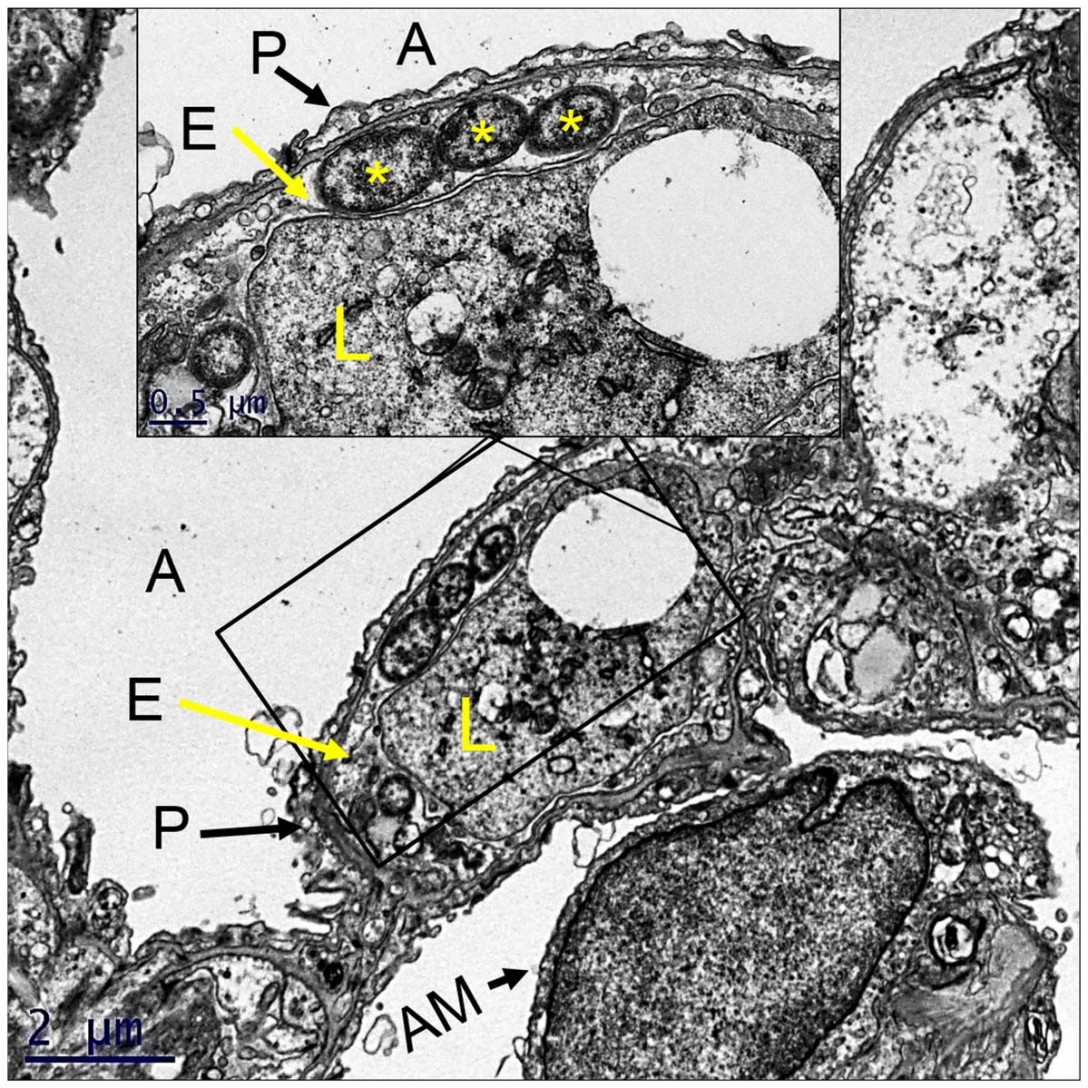
Pulmonary endothelial infection by *Orientia*. Electron micrograph of mouse lung at 6*Orientia*. **Inset** shows higher magnification of cytosolic *Orientia*. A, alveolar space; P, type 1 pneumocyte, E, alveolar capillary endothelial cell; *, three orientiae; L, leukocyte in capillary lumen; AM, alveolar macrophage in adjacent alveolar space.

**Figure 5:**
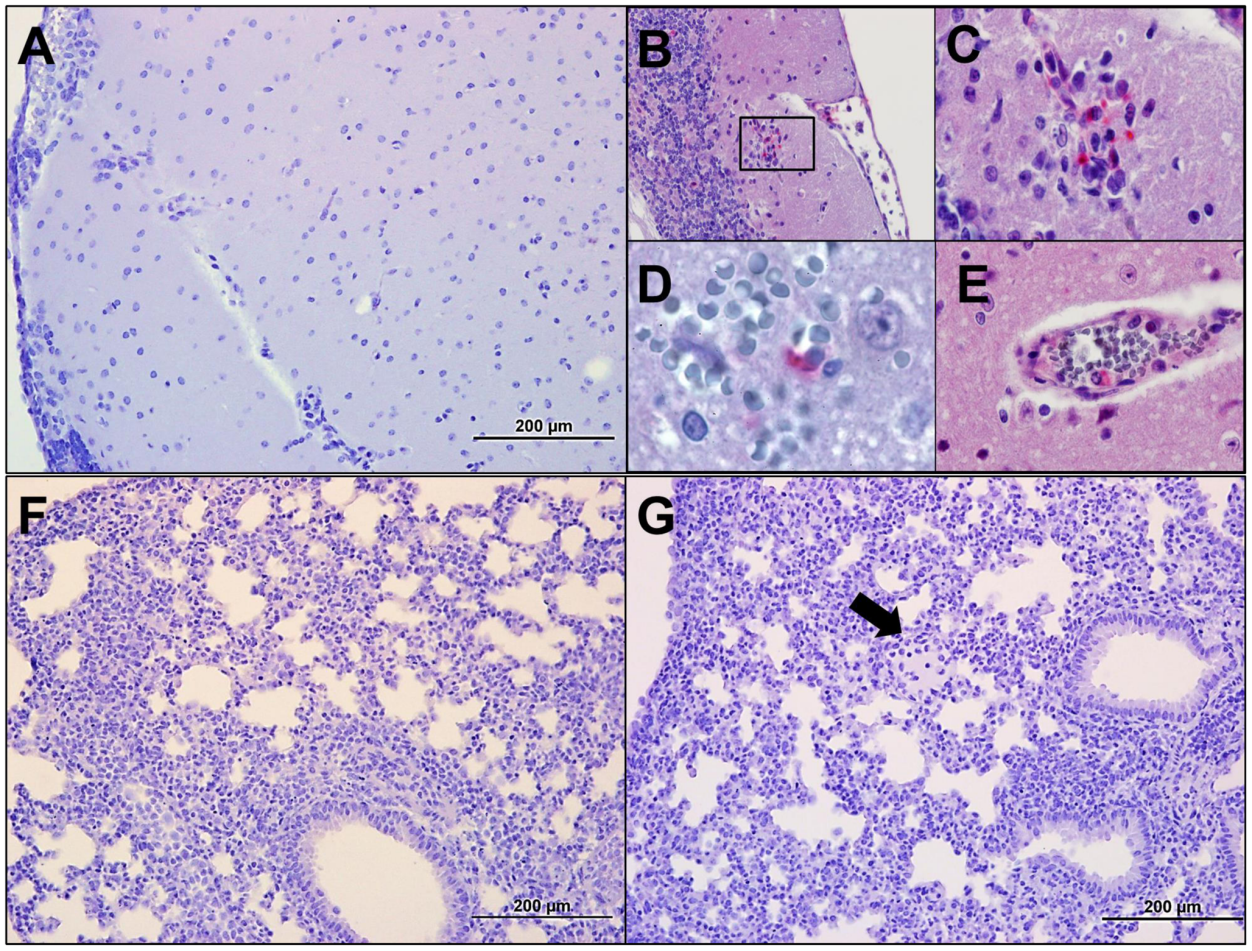
Histopathology of mice following lethal intravenous *Orientia* challenge at days 9 and 12 pi. Immunohistochemical staining of tissues from animals 9 and 12(**A-20×**) was observed in the majority of animals on 9 dpi with cerebral perivascular, lymphohistiocytic infiltrates (**B-inset; 40×, C-100×**), cerebral hemorrhage (**D-100×**), and endothelial infection (**E-100×**) in moribund animals at 11 dpi. Pulmonary cellular infiltrates were marked 9 dpi resulting in interstitial pneumonia (**F-20×**). At 12 dpi (**G-20×**), peribronchial and perivascular cellular infiltration, interstitial pneumonia, and edema (**G-arrow**) were observed in all animals.

**Figure 6:**
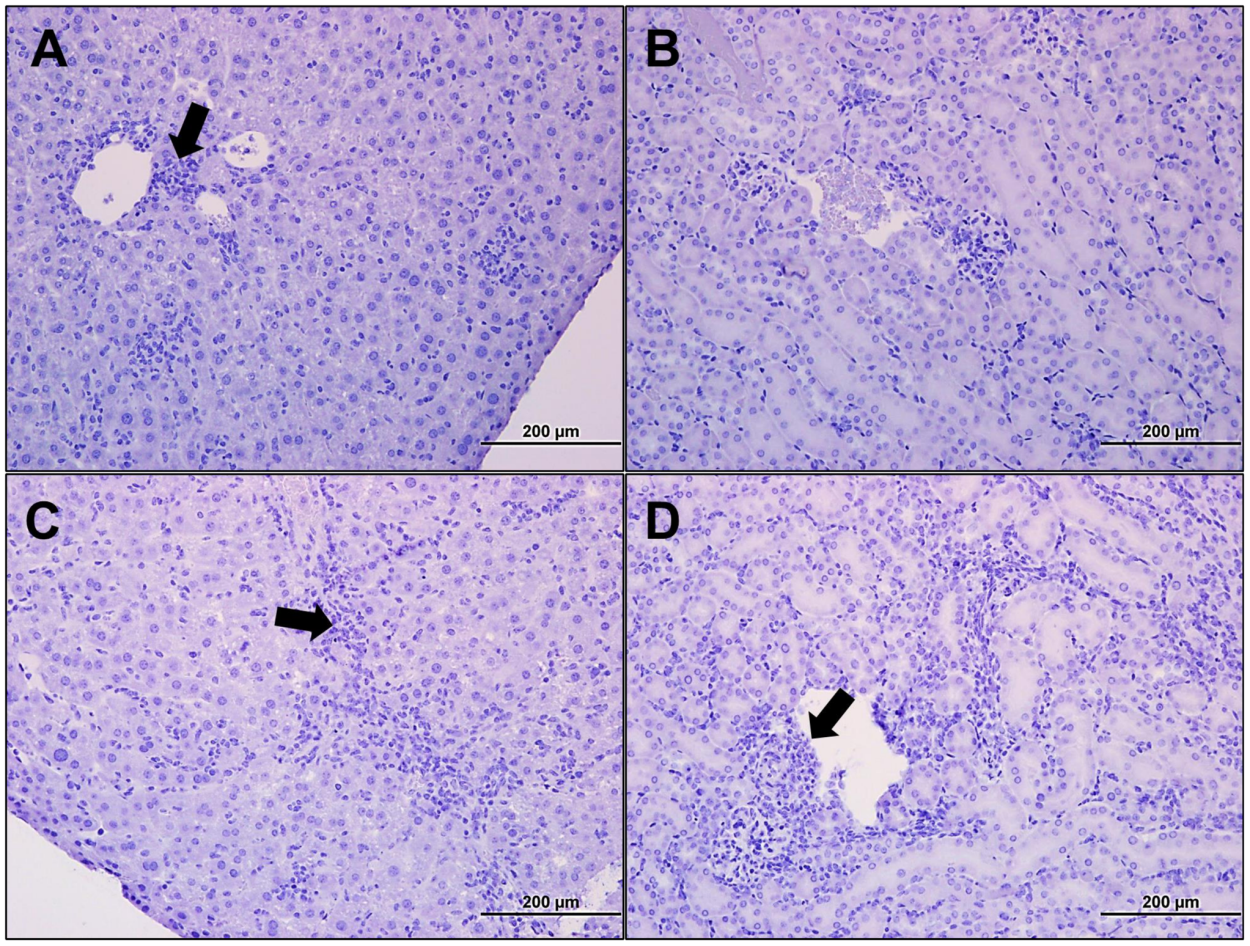
Histopathology of the liver and kidney following lethal intravenous *Orientia* challenge at days 9 and 12 pi. Portal triaditis (**A-arrow; 20×**) was prominent at 9 dpi, and at 12 dpi perivascular infiltrates were observed in the hepatic sinusoids (**B-arrow; 20×**). Mild perivascular infiltrates were observed in the kidney at 9 dpi (**C-20×**), and at 12 dpi (**D-arrow; 20×**) cellular infiltrates were observed throughout the kidney, particularly as peritubular infiltrates.

At necropsy on 3 dpi, i.v. inoculated mice had generalized lymphadenopathy but no other gross lesions. The mice had perivascular lymphohistiocytic infiltrates in the meninges, and *Orientia* antigen was detected in the liver and lung with associated cellular infiltrates in both organs. The kidneys were unremarkable. At 6 dpi, the animals had a slight decrease in body weight (**Figure 1C** **-blue boxes**) and generally appeared healthy although some animals exhibited decreased activity and slightly hunched posture. At this time point, immunohistochemistry demonstrated that systemic infection was established with most of the *Orientia* observed in endothelial cells of the lung (**Figure 3A and B**), kidney (**Figure 3C**), and liver (**Figure 3D**). Endothelial infection was confirmed by electron microscopic analysis of lung sections from i.v infected mice (**Figure 4**). Cellular infiltration had increased at days 9 and 12 (**Figure 5**). Meningitis and cerebral perivascular infiltrates (**Figure 5A, B, C, and E**) were observed on both days, and focal cerebral hemorrhage was observed at 12 dpi (**Figure 5B**). Pulmonary vasculitis and interstitial pneumonia (**Figure 5F and G**) became more severe as the infection progressed. Hepatic (**Figure 6A and B**) inflammatory lesions became more pronounced, and multifocal mononuclear infiltrates were numerous. Cellular infiltrates between the tubules of the kidney (**Figure 6C**) were evident at 9 days post inoculation with renal vasculitis observed at 12 dpi (**Figure 6D**). The pathologic lesions of mice inoculated i.v. became progressively more severe through the course of infection with animals expiring on days 12–13 (**Figure 1A**). Blood, liver, lung, and spleen were monitored for bacterial loads at each time point. At this challenge dose, i.v. and i.p. inoculated animals had detectable bacterial loads in all tissues throughout the course of disease (**Table 1**).

**Table 1:** Bacterial load (47 kDa gene copies/pg of DNA or µL of blood) kinetics of 1.25×10^6^ FFU challenged C57BL/6 mice.

	3 dpi		6 dpi	
	i.v. Mean (Range)	i.p. Mean (Range)	i.v. Mean (Range)	i.p. Mean (Range)
**Blood**	27 (ND^a^-61)	11 (ND^a^-16)	51 (10–110)	71 (51–88)
**Liver**	23,400 (1,290–70,000)	25,470 (2,720–56,800)	9,400 (2,600–21,300)	294,595 (1,270–702,000)
**Lung**	255 (12–823)	360 (40–604)	19,447 (925–14,500)	21,515 (1,130–56,500)
**Spleen**	60,043 (490–219,000)	74,839 (2,260–182,000)	18,925 (1,050–60,200)	12,907 (1,130–44,700)

	9 dpi		12 dpi	
	i.v.	i.p.	i.v.	i.p.
**Blood**	21 (6–35)	27 (ND^a^-48)	11 (8–23)	-
**Liver**	54,566 (942–216,000)	3,854 (168–13,900)	73 (3–197)	-
**Lung**	125,129 (437–45,4000)	267,811 (14,200–1,310,000)	17,323 (3,470–31,200)	-
**Spleen**	31 (11–63)	383,950 (25,800–706,000)	69,096 (185–236,00)	-

a , ND- not detected.

### Circulating blood cell counts

The peripheral blood cell counts of i.p and i.v. inoculated animals were compared to uninfected controls and published normal ranges for B6 mice. At 3 dpi all mice showed slightly elevated WBC counts, mainly neutrophils, compared to uninfected controls, but within the normal range. At day 6, both i.p. and i.v. inoculated animals manifested leukocytosis with lymphopenia, and i.v. inoculated animals had marginally greater elevation of WBC counts than i.p. inoculated animals. Intravenously inoculated animals had neutrophil concentrations three times greater than uninfected controls. At 9 dpi, i.v.-inoculated animals had leukocytosis, mostly neutrophilia but less than on day 6, as well as lymphopenia. Intraperitoneally inoculated animals had lymphopenia and neutrophilia that was less severe than that of i.v. inoculated animals. At 12 days, leukocytosis persisted with neutrophil concentrations being five times greater than uninfected controls. At this time, all i.p. animals had expired (9 dpi), and all i.v. animals were moribund.

### Characterization of the sublethal i.v. *Orientia* infection and same dose i.p. in C57BL/6 mice

**Figure 7:**
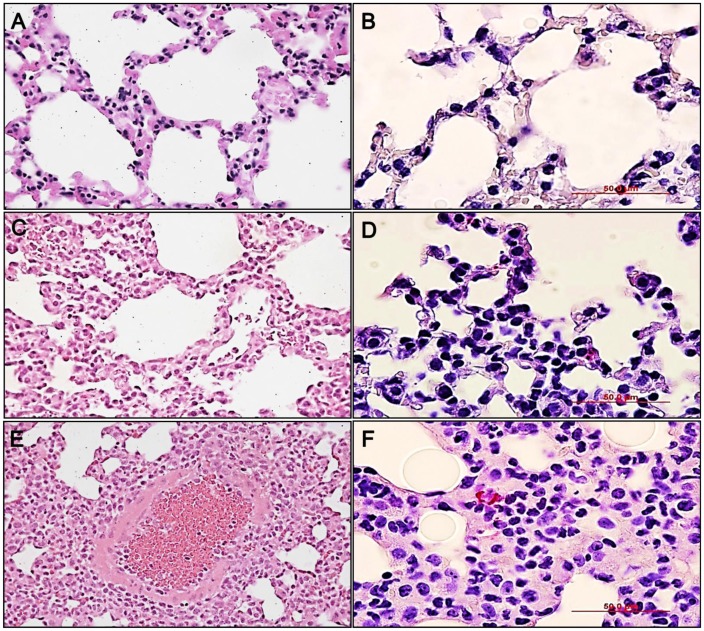
Histopathology of the lung following 1.25×10^4^ *Orientia* challenge. H & E stained uninfected lung tissue (**A-20×**) and IHC of uninfected lung (**B-40×**) compared with lung from i.p. inoculated mice 12 dpi (H&E **C-20×**, IHC **D-40×**), and lung from i.v. inoculated 15 dpi (H&E **E-20×**, IHC **F-40×**). All i.p. infections were lethal with less severe pulmonary cellular infiltrate when compared to that of i.v. infected mice with capillary endothelial cell infection of the aveolar septa.

Disease progression of animals inoculated i.v. with 1.25×10^4^ organisms paralleled that of lethally challenged animals but with signs of illness typically appearing 2–3 days later than in lethally infected animals (**Table 2**). Sublethally infected animals became lethargic and developed severely hunched posture at 12–13 days. With this lower dose, 10% of animals were moribund at 13 days; the remaining animals recovered clinically between 15 and 21 days after infection. Histopathologic observations at the various time points revealed that the lesions progressed similarly to those in the lethally infected animals, but with a dose-dependent delayed onset. Hepatic lesions, mild pulmonary cellular infiltration and *Orientia* antigen were detected at 6 dpi; interstitial pneumonia developed between 9 and 12 dpi. On day 15, multifocal cellular infiltrates were observed in the lungs (**Figure 7**), kidneys, and liver. Animals inoculated i.p. with 1.25×10^4^ *Orientia* organisms had an incubation period two days longer than animals inoculated with the high dose before signs of illness appeared. Unlike the low dose i.v. inoculated animals, this dose administered i.p. was uniformly lethal by day 15 (**Figure 1B**). Severe peritonitis was observed with accumulation of peritoneal exudate in excess of 2 mL. *Orientia* antigen was detected focally in the lungs in association with vasculitis and interstitial pneumonia on day 15. At this time point, the animals' body weights had increased, owing to the accumulation of exudate in the peritoneal cavity. Bacterial loads of animals inoculated with *Orientia* were monitored at each time point. At this challenge dose, i.v. and i.p. inoculated animals had detectable bacterial loads in liver, lung, and spleen throughout the disease course (**Table 3**). *Orientia* in the blood was not detected consistently until 6 dpi for i.v. inoculated animals and 9 dpi for i.p. inoculated animals. All surviving animals began to recover weight, and signs of illness resolved (**Figure 1D**).

**Table 2:** Summary of disease manifestations.

	1.25E+06		1.25E+04	
Pathology/Route of Inoculation	IV	IP	IV	IP
Survival	0/10	0/10	9/10	0/10
Onset of illness*	6 dpi	5 dpi	8 dpi	7 dpi
Day of Death	10–13 dpi	8–9 dpi	13 dpi	10–15 dpi
Abdominal swelling	0/10	7**/10	3***/10	10**/10
Peritonitis	0/10	7/10	0/10	10/10
Pleural effusion	7/10	0/10	10/10	3/10
Hepatomegaly	10/10	10 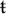 /10	10/10	10 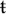 /10
Splenomegaly	10/10	10 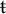 /10	10/10	10 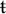 /10
Peripheral lymphadenopathy	10/10	8/10	10/10	10/10
Mesenteric lymphadenopathy	2/10	10/10	5/10	10/10

*, mice began exhibiting hunched posture, lethargy, ruffled fur, and rapid breathing.

**, Animals had accumulation of turbid, peritoneal exudate.

***, Animals had accumulation of ascites fluid.

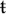
, Hepatosplenomegaly was less severe than i.v. inoculated animals.

**Table 3:** Bacterial load (47 kDa gene copies/pg of DNA or µL of blood) kinetics of 1.25×10^4^ FFU challenged C57BL/6 mice.

	3 dpi		6 dpi	
	i.v. Mean (Range)	i.p. Mean (Range)	i.v. Mean (Range)	i.p. Mean (Range)
**Blood**	3 (ND^a^-4)	ND^a^	92 (ND^a^-170)	6 (ND^a^-11)
**Liver**	32 (4–67)	28 (ND^a^-47)	558 (61–1,380)	2,934 (80–6,530)
**Lung**	18,140 (4,120–48,700)	671.18 (ND^a^-2,100)	446,975 (18,100–1,220,000)	46,005 (1,610–115,000)
**Spleen**	1,466 (199–3,470)	16,275 (ND^a^-24,000)	9,875 (1,400–27,500)	26,375 (9,400–41,400)

	9 dpi		12 dpi	
	i.v.	i.p.	i.v.	i.p.
**Blood**	408 (ND^a^-779)	15 (5–23)	13 (ND^a^-43)	88 (12–158)
**Liver**	1,501 (124–2,860)	14,400 (421–46,200)	70 (18–190)	1,716 (334–3,590)
**Lung**	525,625 (28,500–1,020,000)	133,425 (21,400–275,000)	24,215 (4,380–64,600)	97,593 (2,290–340,000)
**Spleen**	7,100 (4,360–11,300)	66,008 (5,530–165,000)	2,601 (422–5,770)	38,720 (3,680–66,900)

	15 dpi	
	i.v.	i.p.*
**Blood**	ND^a^	ND^a^
**Liver**	92 (ND^a^-348)	4 (3.48–3.53)
**Lung**	5,398 (141–18,000)	846 (561–1,130)
**Spleen**	8,149 (107–30,300)	169 (109–228)

a , ND- not detected.

*, only two animals remained.

## Discussion

Scrub typhus has been described as one of most severely neglected tropical diseases; indeed it has potentially more fatal cases annually than dengue fever [3]. It was first described in China in 84 B.C. and made its presence felt during the wars that took place in the region during the last century.

Animal model development is important for understanding pathogenesis and immunity and for preclinical testing of vaccines and therapeutics. Accurate animal models for diseases are imperative to developing sound understanding of the diseases. Models that do not present similar features as the human disease may provide misleading information about the disease, further impeding the understanding of disease progression. In the case of scrub typhus, the intraperitoneally inoculated mouse model that has been used for the last 60 years results in severe peritonitis, a condition that does not occur in human scrub typhus, yielding an inappropriate model for this disease.

Studies of immunity, primarily in intraperitoneally inoculated mice, were performed more than 25 years ago when many contemporary tools and concepts of immunology had not been developed. The presently developed model will enable valid determination of the mechanisms of protective immunity according to contemporary concepts of immunology in an animal system that accurately models human scrub typhus.

The i.v. inoculation of *O. tsutsugamushi* Karp strain resulted in a hematogenously disseminated scrub typhus model that reliably produced pathology and target cell tropism similar to scrub typhus in humans. Similar to other members of the Rickettsiaceae family, *Orientia* predominantly infects endothelial cells after dissemination from the site of mite feeding [7]. How this occurs remains to be elucidated. The intravenously infected animals developed disseminated endothelial infection and histopathology as occurs in human scrub typhus. Both B6 and C3H mice exhibited similar disease course when challenged with Karp strain, with C3H mice being marginally more susceptible than B6 mice. Both of these mouse strains have been used in scrub typhus research as well as the study of spotted fever group rickettsioses [20]–[24]. B6 mice were chosen to fully characterize the histopathology and disease course of the i.v. model due to the abundant conditional and gene knockout strains on the B6 background for use in future studies.

Although *Orientia* disseminated after intraperitoneal inoculation, the resulting peritonitis that occurs following this mode of infection, which does not occur in human scrub typhus, was the dominant pathological feature. The tissue bacterial loads observed during this study demonstrate that the route of inoculation is pivotal in the development of scrub typhus-like pathology. Both routes of inoculation result in bacterial dissemination (**Table 1** and **2**), but intravenous inoculation avoids stimulating the immune response of the peritoneal cavity and thus does not generate the lethal peritonitis observed in i.p. inoculated animals. The lower dose animals had similar bacterial distribution as the high dose animals for both routes of inoculation (**Table 2**), but the associated pathology also developed in an inoculation route-dependent manner. The oriential antigen at time of death in i.p. inoculated animals was observed predominantly on the peritoneal surface of the liver and spleen (**Figure 2**) while oriential antigen in i.v. inoculated animals was predominantly in endothelial cells (**Figures 3** **–** **6**).

The pathology of scrub typhus is characterized by multifocal cellular infiltrates around the blood vessels of all organs, particularly the brain, lungs, and liver. The central nervous system (CNS) is frequently involved in scrub typhus infection. Headache, nausea, vomiting, transient hearing loss, confusion, neck stiffness, delirium, and mental changes may be observed [26]. Glial nodules consisting of perivascular infiltration by lymphocytes and macrophages in the neuropil as well as perivascular hemorrhage were observed in our lethal model; those findings strongly resemble the lesions and cell tropism described in humans by Allen and Spitz (1945) and Moron *et al.* (2001) [7], [11].

Respiratory involvement is common in severe scrub typhus infections. Approximately 40% of scrub typhus patients manifest cough at the time of admission [27]. Interstitial pneumonia, pulmonary edema, pleural effusions, cardiomegaly, and/or focal atelectasis are observed by chest radiography in those patients [10], [28]. The presence of respiratory symptoms is closely linked to severity of scrub typhus [29]. Pulmonary pathology observed in humans comprises interstitial pneumonia with mononuclear cell infiltrates [7], [11], [30]. The intravenously infected animals developed similar lesions.

Hepatomegaly and modest elevations of serum aminotransferases have been documented in humans. Those laboratory abnormalities might be associated with pathological changes in the liver similar to those described here in the intravenous mouse model of fatal scrub typhus [7], [11], [31].

Severe scrub typhus frequently results in acute renal failure [32]. Cellular infiltrates around the microvasculature of the kidney, particularly between the tubules, were observed by Allen and Spitz (1945) [11]; similar findings were prominent during later time points in the mouse model described here.

The ideal model would involve an animal closely related to humans, i.e., nonhuman primates (NHP), and mite transmission, but both of these aspects would be difficult to obtain, both from an expense and expertise point-of-view. NHPs are expensive, and acquiring the number required to characterize the basic immunology and histopathology to validate the model would be cost prohibitive. Early studies conducted using NHPs had difficulty finding individuals that had not been exposed in nature to *Orientia* prior to experimentation [33]–[38]. The most recent studies to use NHPs focused on temperature and weight changes as clinical indicators of disease and did not obtain histopathologic evidence that NHPs develop pathology similar to humans; in fact, lethal infections were not achieved after i.d. inoculation [39], [40]. Secondly, very few mite colonies exist, with most containing multiple strains of *Orientia* [41], which would make the study of the immune response during infection even more challenging. Only i.d. inoculation of *Orientia* into NHP results in eschar-like lesions [39], [40].

The use of mice as a model, especially C57BL/6 mice, provides many advantages including the availability of reagents and the availability of gene and conditional gene knockout strains on the B6 background. This model will allow us to study lethally and sublethally challenged animals to determine the factors that play a role in severe disease and using knockouts, adoptive transfers, or other methods to modulate the immune response to increase survival and decrease disease severity.

There have been articles published recently addressing model development for scrub typhus [42], [43]. The i.d. inoculation of *Orientia* by mites, and the subsequent immune response to this event, is an important step in the infection. Mite transmission resulted in variably lethal infection of the outbred mice studied, and the time to death after disease onset was 5–9 dpi which is similar to our model [42]. The published i.d. inoculation study only followed the mice for 7 dpi and only examined the dissemination of different strains of *Orientia*. Data were not provided to compare the entire clinical course and development of systemic pathology for these models [43]. The i.v. model aims to simulate scrub typhus once the bacteria have left the eschar and begin to systemically infect the endothelium. The histopathology of mite transmission and i.d. inoculation models has not been thoroughly characterized; thus, comparison to the i.v. model's histology is not possible at this time.

The clinical signs of our mice were similar to those described in the mite transmission model, with weight loss and decreased activity preceding death [41]. Bacterial dissemination was not followed in the mite transmission model, and thus it is impossible to compare this feature to our model, but the i.d. inoculation model did show remarkably rapid dissemination from the site of inoculation into the lungs as early as 24 hours post infection [42]. In the i.v. model, orientiae were detected at every time point in the majority of animals tested with a peak on day 6 post infection. How these bacterial kinetics compare to those of the i.d. inoculation model is not known as data throughout the disease course are not available.

In conclusion, the model characterized in this study closely parallels the clinical course and pathological lesions described for lethal scrub typhus in humans. Intravenous inoculation of 1.25×10^6^ *Orientia* resulted in an acute infection that culminated in death at 12–13 dpi. Pathological progression was observed in animals euthanized at sequential time points during the course of illness. With the establishment of lethal and sublethal doses for the intravenous model of scrub typhus, it will be possible to begin elucidating, mechanistically, the host responses that result in lethal outcomes or in protective immunity. As this model was established using the C57BL/6 mouse strain, future research projects will be able to utilize the abundant gene knockout mouse strains available on this background to determine the role of specific cell types and immune components involved in scrub typhus immunity and pathogenesis. The development of this model will provide a powerful tool to characterize the immunology of scrub typhus infection and a relevant model for vaccine testing that is intended to lead to an effective vaccine that produces long lasting immunity.
