# Correction: A Hematogenously Disseminated *Orientia tsutsugamsushi* -Infected Murine Model of Scrub Typhus

**DOI:** 10.1371/journal.pntd.0003175

**Published:** 2014-08-25

**Authors:** The PLOS Neglected Diseases Staff

The word “*tsutsugamushi*” is misspelled in the article title. The correct title is: A Hematogenously Disseminated *Orientia tsutsugamushi*-Infected Murine Model of Scrub Typhus. The correct citation is: Shelite TR, Saito TB, Mendell NL, Gong B, Xu G, et al. (2014) A Hematogenously Disseminated *Orientia tsutsugamushi*-Infected Murine Model of Scrub Typhus. PLoS Negl Trop Dis 8(7): e2966. doi:10.1371/journal.pntd.0002966

## References

[1] Shelite TR, Saito TB, Mendell NL, Gong B, Xu G, et al (2014) A Hematogenously Disseminated *Orientia tsutsugamsushi*-Infected Murine Model of Scrub Typhus. PLoS Negl Trop Dis 8(7): e2966 doi:10.1371/journal.pntd.0002966 25010338 10.1371/journal.pntd.0002966 PMC4091938

